# Late Adolescents’ Attachment to Parents and Peers and Psychological Distress Resulting from COVID-19. A Study on the Mediation Role of Alexithymia

**DOI:** 10.3390/ijerph182010649

**Published:** 2021-10-11

**Authors:** Renata Tambelli, Silvia Cimino, Eleonora Marzilli, Giulia Ballarotto, Luca Cerniglia

**Affiliations:** 1 Department of Dynamic and Clinical Psychology, Sapienza University of Rome, 00186 Rome, Italy; renata.tambelli@uniroma1.it (R.T.); eleonora.marzilli@uniroma1.it (E.M.); giulia.ballarotto@uniroma1.it (G.B.); 2 Faculty of Psychology, International Telematic University Uninettuno, 00186 Rome, Italy; l.cerniglia@uninettunouniversity.net

**Keywords:** late adolescence, attachment, alexithymia, COVID-19, peritraumatic distress

## Abstract

The scientific literature has shown the key role played by attachment to parents and peers and difficulties in recognizing, processing, and regulating emotions (i.e., alexithymia) in the (mal-)adaptive psychological response to the COVID-19 pandemic during late adolescence. No study has yet explored the complex interplay between these variables. We recruited a sample of 454 late adolescents (Mage = 22.79, SD = 2.27) and assessed attachment to parents and peers, alexithymia, and peritraumatic distress due to COVID-19 through self-report instruments. Attachment to fathers and peers, but not to mothers, and alexithymia significantly predicted levels of peritraumatic distress. Alexithymia fully and partially mediated the effect of, respectively, attachment to mothers and attachment to peers on peritraumatic distress due to COVID-19. These findings suggested that intervention programs focused on the promotion of peer social relationships, supportive parent–adolescent relationships, and the ability to recognize and discriminate one’s own and others’ emotions are needed in helping late adolescents to face the current health emergency and preventing short- and long-term psychopathological consequences related to the COVID-19 pandemic.

## 1. Introduction

The COronaVIrus Disease 19 (COVID-19) emerged in Wuhan, China, in late December 2019. Its rapid diffusion across the world and the related measures put in place to stem its spread have had a crucial impact on the global economy, as well as on the daily habits and quality of life of people [1], especially young people’s lives [2]. Some individuals have experienced a sense of efficacy and managed to adapt to the new circumstances imposed by the COVID-19 restrictions [3], manifesting an adaptive response to the COVID-19 pandemic, but a large part of the general population has shown maladaptive psychological responses, reporting psychopathological symptoms, including anxiety [4,5], depression [6,7], and post-traumatic stress symptoms [8,9,10]. Although most studies have focused on adult samples of the general population, relevant consequences of COVID-19 on late adolescents’ mental health have also been shown [7,11,12].

The developmental phase of “late adolescence/emerging adulthood” (between the ages of 18 and 25) [13,14,15] represents a developmental period particularly at risk for the psychological short- and long-term consequences of COVID-19 pandemic and its related restrictions. Clinicians and developmental researchers have long referred to youths aged from 18 to 25 years old only in terms of “emerging adults” [13] or “young adults” [16], highlighting the exit from adolescence and entry into adulthood. However, recent works have suggested the importance of expanding the definition and timeframe of adolescence, including youths up to about 25 years of age, to more closely reflect current patterns of adolescent growth [15,17]. In line with this suggestion, contemporary scientific literature refers to this specific age range also using more inclusive terms, such as “late adolescence” [18,19] or “youth” [20,21]. From a developmental point of view, late adolescents and young adults must face important evolutionary tasks (i.e., the acquisition of identity, autonomy, and self-determination; the reorganization of the relationships with parents and peers) [22], and some studies have suggested that many containment measures to prevent the COVID-19 spread (e.g., the switch to distance education, social distancing, the increase in closure of playgrounds and recreational spaces) may have made these evolutionary steps even more difficult [12,23,24]. Youths had to spend much more time at home with their parents during a phase of life in which, physiologically, they tend to prefer time spent with their peers and to become more independent from families [25,26], with a significant impact on adolescents’ psychological well-being [27,28]. Consequently, given the clinical relevance of the phenomenon, further studies aimed at identifying possible risk and protective factors associated with young adults’ psychological maladaptive response to the COVID-19 pandemic are needed to implement the planning of targeted prevention programs and interventions. In this field, the Developmental Psychopathology theoretical framework [29,30] considers an individual’s developmental outcomes as the result of interactions between risk and/or protective factors of different domains. Specifically, clinicians and researchers who work from this perspective have shown the importance of considering the role played by adolescents’ individual characteristics (i.e., personality traits) [31,32,33,34] and relational factors (i.e., the quality of the relationship with parents and peers) [35,36], as well as their dynamic relationship in studying psychopathological difficulties in response to a stressful life event (such as the COVID-19 pandemic).

Among relational factors, the quality of attachment to parents and peers can represent a crucial risk and/or protective factor that may promote or mitigate adolescents’ psychological consequences of an adverse experience [37,38]. Indeed, one of the main functions of attachment is to regulate distress in times of exposure to stress situations [39,40], as the COVID-19 outbreak. In this field, studies have shown that youths with secure attachment showed more adaptive strategies when faced with a fearful situation, tending to seek comfort and emotional support from parents and friends [41,42]. Conversely, insecure attachment adolescents tend to use maladaptive responses in facing a stressful life event [43,44], showing higher anxiety levels [45], and post-traumatic stress disorder symptoms [46]. Recently, studies have shown that the quality of attachment may also influence individuals’ experience of fear during the COVID-19 pandemic and the resulting psychopathological outcomes [44,47,48,49,50,51]. Specifically, the COVID-19 outbreak can be considered as a stress inducer [52] that may active attachment behavior strategies and the related individual’s emotional and behavioral responses used in previous stressful situations [51]. Coherently, significant associations between insecure attachment and higher psychological distress have been reported [48,50,51]. However, very few studies have focused on the adolescent population [49], and no study has yet explored the possible specific contribution played by, respectively, late adolescents’ attachment to parents and peers.

Beyond the role played by relational factors, a key role of youths’ emotional difficulties (i.e., high alexithymic traits) has also been highlighted in the context of the psychopathological impact of stressful life events [53,54,55,56]. Alexithymia refers to an individual’s difficulties in recognizing, processing, and regulating emotions [57]. Consequently, a late adolescent with difficulties in identifying one’s feelings may be at higher risk in the face of a stressful experience, due to deficiencies in symbolically thinking [58] and in processing and regulating his/her emotions and the related responses [59,60]. Recently, the predictive effect of alexithymic traits on psychopathological symptoms resulting from the COVID-19 pandemic has also been reported [61,62,63,64]. Interestingly, international research has widely shown that insecure attachment exerted a significant contribution in individuals’ emotional regulation difficulties [65,66], predisposing adolescents to higher levels of alexithymic traits [67,68]. Moreover, recent studies have shown the complex relationship between individuals’ attachment, alexithymia, and psychopathological difficulties, showing that adolescents’ alexithymic traits played a mediation role in the effect of attachment on psychopathological difficulties [69,70]. Overall, findings from international literature have shown the predictive role of attachment on levels of alexithymia [67,68], that in turn predicted psychopathological outcomes resulting from COVID-19 [61,62,63,64]. This evidence suggests that adolescent’s attachment could influence psychological responses to the COVID-19 pandemic more directly than via alexithymia. However, to date, no study has yet explored whether late adolescents’ alexithymia could mediate the relationship between attachment to parents and peers and psychological distress due to COVID-19.

Based on the above premises and literature gaps, the present study aimed to verify, in a community sample of late adolescents, the possible complex relationship between attachment to mothers, fathers, and peers, alexithymia, and psychopathological symptoms resulting from the pandemic. Specifically, the study aimed to address the following research questions: (1) Does late adolescents’ attachment to parents and peers, and levels of alexithymia, have an impact on peritraumatic distress due to COVID-19? In line with previous studies [67,68], we hypothesized that adolescents’ attachment to parents and peers significantly predicted high levels of alexithymia; (2) Does adolescents’ attachment to parents and peers indirectly affect adolescents’ peritraumatic distress due to COVID-19 via adolescents’ alexithymia? Based on recent studies showing that individuals’ alexithymia significantly mediated the relation between attachment and psychopathological outcomes [69,70], we hypothesized to find a significant mediation role of late adolescents’ alexithymia also on effects exerted, respectively, by attachment to parents and peers on levels of peritraumatic distress resulting from COVID-19.

## 2. Materials and Methods

### 2.1. Sample, Recruitment, and Procedure

The study was conducted between 15 November 2020 and 15 March 2021, during the Italian second wave of COVID-19. We recruited N = 642 late adolescents (age range between 18 and 25 years) via social media. After giving their written agreement to participate, each participant filled out an anonymous online survey composed of self-report instruments (described below) assessing the quality of adolescents’ attachment to mothers, fathers, and peers, alexithymia, and peritraumatic distress resulting from the COVID-19 pandemic.

From the total sample, we excluded late adolescents who did not complete the assessment procedure (*n* = 113), who reported psychiatric diagnoses and/or physical disorders (*n* = 34), and who were following psychological and/or psychiatric treatment (*n* = 41). The final sample consisted of N = 454 late adolescents (57.3% females) with average age of 22.79 (SD = 2.27). Subjects most often reported their highest level of education being high school (43%) or more than high school (54.2%), and 55.9% were students without a job (55.9%). The majority (59%) were single, 75.1% had married or cohabiting parents (i.e., intact families), and most of them (78.9%) lived within the family members.

Before the start of the study, the research plan was approved by the Ethical Committee of the Department of Dynamic and Clinical Psychology at Sapienza University of Rome (protocol N. 809/2020), in accordance with the Declaration of Helsinki.

### 2.2. Measures

All late adolescents who decided to participate in the study filled out the Inventory of Parent and Peer Attachment (IPPA) [71,72], the COVID-19 Peritraumatic Distress Index (CPDI) [73,74], and the Toronto Alexithymia Scale (TAS-20) [75,76] for the assessment of the variables under study.

The IPPA [71] is a self-report questionnaire used for the assessment of adolescents’ perception of the quality of relationships with their mothers, fathers, and peers, considered in terms of feelings of security and positive/negative aspects of these relationships. It is composed of three parts that included items measured on a five-point Likert-scale response format, respectively, related to the attachment to mothers (composed of 28 items), fathers (composed of 28 items), and peers (composed of 25 items). Higher scores are indicative of greater attachment security. Italian validation [72] showed good psychometric proprieties (Cronbach’s alpha ranging from 0.62 to 0.90).

The CPDI [73,74] is a self-report instrument for the assessment of psychological distress resulting from COVID-19. Specifically, the CPDI is composed of 24 items measuring a series of symptoms related to the criterion A of PTSD (e.g., anxiety, depression, phobias, avoidance behaviors, compulsive behaviors, and loss of social functioning). The total score ranges from 0 to 100. Higher scores are indicative of more psychological distress. Studies have shown a good internal coherence of CPDI [73,74].

The TAS-20 [75,76] is a self-report questionnaire used for the assessment of alexithymia. It is composed of 20 items measured on a 5-point Likert scale (from 1 = strongly disagree, to 5 = strongly agree). The scale is composed of three factors, assessing the ability to recognize emotions, to describe verbally one’s own emotions, and the tendency of externally oriented thinking. The sum of scores of the three factors provides a Total score, used in this study. Higher scores indicate higher levels of alexithymia. The TAS-20 showed good internal consistency and test–retest reliability (Cronbach’s alpha of the total score is 0.86).

### 2.3. Statistical Analyses

First, preliminary statistical analyses were carried out (reliability of the measures, frequencies, mean scores, and percentages). Then, after verifying normality of distribution and linearity, we conducted Pearson’s correlation analyses to determine significant correlations between study variables and to identify significant sociodemographic covariates. Based on significant correlations that emerged, hierarchical multiple regression analyses were carried out to identify the main effects of adolescents’ attachment to parents and peers, and levels of alexithymia on adolescents’ peritraumatic distress due to COVID-19 pandemic, controlling for relevant covariates. We preliminary tested for homoscedasticity and multicollinearity of data. Finally, mediation analyses were conducted to verify whether adolescents’ levels of alexithymia mediated the effect, respectively, of adolescents’ attachment to mothers, fathers, and peers on adolescents’ peritraumatic distress due to COVID-19 pandemic. Consequently, three separate mediation models were conducted. Indirect effects were evaluated with 95% bias-corrected confidence intervals (CIs) based on 10.000 bootstrap samples. All analyses were performed using IBM SPSS software, version 26.0. Mediation analyses were conducted used Hayes’s PROCESS macro [77] (Model 4).

## 3. Results

### 3.1. Correlations between Study Variables

Table 1 shows the results of Pearson’s correlation analyses between sociodemographic variables (i.e., adolescent’s sex, age, educational level, occupation, relationship status, family status, and living setup), adolescents’ peritraumatic distress due to COVID-19, adolescents’ attachment to mothers, fathers, and peers, and levels of alexithymia. In particular, adolescents’ peritraumatic distress due to COVID-19 was significantly and negatively associated whit adolescents’ attachment to mother, father, and peer, and positively associated with levels of alexithymia.

Moreover, higher levels of adolescents’ alexithymia were negatively associated with attachment to mother, father, and peers, supporting the prerequisites for the hypothesized mediation role played by alexithymia on the relationship between adolescents’ attachment to parents and peers and peritraumatic distress due to COVID-19. Finally, all sociodemographic variables considered were related to many of the study variables (except for adolescents’ sex), and were included as covariates in subsequent analyses.

### 3.2. Main Effects of Adolescents’ Attachment to Parents and Peers, and Levels of Alexithymia on Peritraumatic Distress Due to COVID-19

Based on significant correlations that emerged, we conducted hierarchical multiple regression analyses to explore whether adolescents’ attachment to mothers, fathers, and peers, and their levels of alexithymia were predictive of levels of adolescents’ peritraumatic distress due to COVID-19, controlling for relevant sociodemographic covariates. As possible to see in Table 2, the scores of attachment to father and peers were significantly predictive of lower adolescents’ peritraumatic distress due to COVID-19. Conversely, high levels of adolescents’ alexithymia were predictive of high peritraumatic distress due to COVID-19. The relationship between attachment to mother and peritraumatic distress due to COVID-19 was not significant. Finally, living setup was confirmed to be a significant covariate and was inserted as a covariate in mediation analyses. The model accounted 31% of the variance. Table 3 shows results of model ANOVA.

### 3.3. Late Adolescents’ Alexithymia as Mediators of the Relationship between Attachment to Parents and Peers and Peritraumatic Distress Due to COVID-19

**Figure 1:**
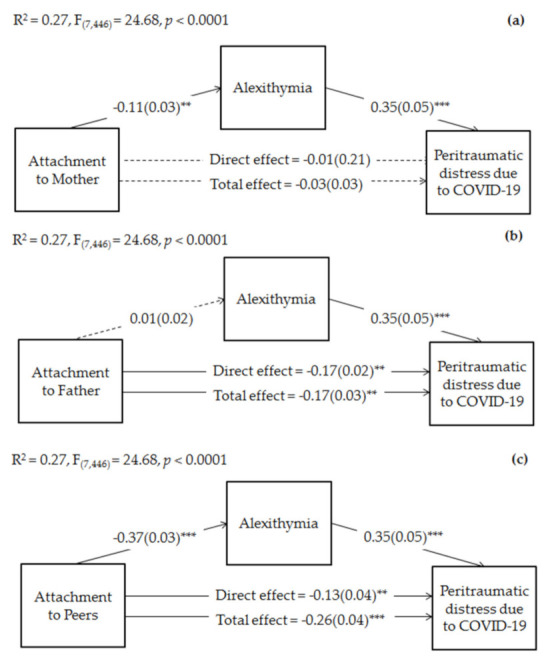
Mediation of late adolescents’ alexithymia on the relationships between attachment to mothers and peritraumatic distress due to COVID-19 (**a**), attachment to fathers and peritraumatic distress due to COVID-19 (**b**), and attachment to peers and peritraumatic distress due to COVID-19 (**c**). Coefficients shown are standardized path coefficients. Dotted lines represent non-significant parameters. c’ = direct effect; c = total effect. ** *p* < 0.01, *** *p* < 0.001.

Finally, we conducted mediation analyses to verify whether adolescents’ levels of alexithymia mediated relationships between attachment to mothers, fathers, and peers with adolescents’ peritraumatic distress due to COVID-19. In each mediation model, we inserted living setup and, respectively, attachment to fathers and peers, attachment to mothers and peers, and attachment to mothers and fathers, as covariates. As possible to see in Figure 1a, results of mediation analyses showed that the total and direct effects of adolescents’ attachment to mothers on adolescents’ peritraumatic distress due to COVID-19 were not significant. However, attachment to mothers significantly and negatively predicted high levels of adolescents’ alexithymia, that in turn significantly predicted high levels of peritraumatic distress due to COVID-19. On the other hand, the total and direct effects of attachment to fathers (Figure 1b) and attachment to peers (Figure 1c) on adolescents’ peritraumatic distress were both significant. However, when considering the effect of the mediator, the coefficient of the direct effect of attachment to fathers remained of the same size, whereas the coefficient of attachment to peers become smaller. Finally, attachment to fathers was not significantly associated with adolescents’ levels of alexithymia, whereas attachment to peers was a significant negative predictor. Overall, each model explained 27% of the variance in adolescents’ peritraumatic distress due to COVID-19.

Regarding indirect effects, as possible to see in Table 4, alexithymia significantly mediated the relationship between attachment to mother and adolescents’ peritraumatic distress, suggesting its full mediation role. Moreover, the indirect path of attachment to peers on peritraumatic distress via alexithymia was also significant.

## 4. Discussion

This study aimed to further increase the knowledge on the complex interplay between youths’ attachment to parents and peers, alexithymia, and psychopathological outcomes related to the COVID-19 pandemic in late adolescence. Specifically, we have chosen to verify the possible role played by attachment to parents and peers, and alexithymia, based on previous literature showing their key contribution in adolescents’ psychological responses and mal-adjustment in the face with stressful life events [53,54,55,56]. To date, only few studies have explored these associations in the specific context of late adolescents/young adults’ peritraumatic distress due to COVID-19 [61,63]. In addition, to the best of our knowledge, no study has yet explored possible indirect effects of attachment to mothers, fathers, and peers via late adolescents’ alexithymia on psychological distress resulting from COVID-19, that this study hypothesized to find. Overall, our findings are in line with our hypothesis.

### 4.1. Main Findings

Regarding possible main effects exerted by late adolescents’ attachment to parents and peers and alexithymia on peritraumatic distress due to COVID-19, results of hierarchical multiple regression analyses showed significant predictions of attachment to fathers and peers (but not to mothers), and levels of alexithymia. Specifically, controlling for relevant sociodemographic factors, late adolescents’ attachment to fathers and peers were negatively associated with adolescents’ peritraumatic distress due to COVID-19, whereas there was a significant positive association with high levels of adolescents’ alexithymia.

These results are in accordance with recent studies showing higher psychopathological symptoms resulting from the COVID-19 outbreak among individuals with insecure attachment [48,49,50,51]. Late adolescence represents a critical phase of life marked by instability and feelings of anxiety and depression [78], and a risk window for the onset of psychopathological problems [79,80,81]. The quality of attachment to parents assumes a crucial role in mitigating or exacerbating this risk [82,83,84], especially in response to stressful experiences. Indeed, stressful life events—as the COVID-19 pandemic—activate the attachment system [39,40], leading the adolescent to activate the related coping strategies and emotional responses in the face of fearful experience [51]. Specifically, in accordance with the Attachment Diathesis-Stress Process Model [85,86], attachment insecurity represents a diathesis that may lead to a maladaptive response when facing stressful life experiences, influencing how the individual perceives the stressful stimulus and subsequent behaviors of seeking support and reassurance from attachment figures. Consequently, individuals characterized by insecure attachment could have more difficulties in sharing their emotions and feelings resulting from the COVID-19 pandemic than secure ones [49,50]; in the presence of dangerous and stressful situations, insecure youths tend to exhibit a lower sense of self-efficacy [87,88] and are less confident of receiving closeness and seeking emotional support from their social environment [89,90], with higher psychopathological outcomes than their secure peers [37,38]. During late adolescence, extrafamiliar relationships become more central in individuals’ affective life [25,26,91]. The research has shown that late adolescents who have attachment insecurity to friends showed higher psychopathological symptoms than their secure peers, whereas attachment security represents a protective factor [70,92]. Nevertheless, although a higher psychopathological risk among insecure adolescents has also been reported in the context of COVID-19 [49], our study is the first to explore the possible specific role played by attachment to mothers, fathers, and peers, showing a significant contribution exerted by late adolescents’ attachment to fathers and peers. Unexpectedly, attachment to mothers was not significantly associated with late adolescents’ peritraumatic distress due to COVID-19, although previous studies have widely shown the key role played by the quality of mother-child relationship in shaping offspring’s psychopathological difficulties overtime [68,93,94,95]. However, it is important to note that results of our preliminary correlation analyses have shown a significant association between late adolescents’ attachment to mothers and alexihtymia, suggesting a possible indirect effect of attachment to mother on peritraumatic distress due to COVID-19 via late adolescents’ alexithymia (a result discussed below).

Regarding the specific role played by late adolescents’ alexithymia on the psychological impact of COVID-19, our results have confirmed that higher levels of alexithymia are predictive of late adolescents’ emotional-behavioral difficulties [96,97,98]. This could be due to the difficulties in regulating and identifying own and others’ feelings [58,59,60], which would expose the late adolescent to greater vulnerability in coping with stressful situations [53,54,55,56].

In this field, as suggested by Dincer, Ayaz, and Oğuz [99], the increased levels of stress and anxiety experienced during the pandemic, together with reduced sharing of emotions due to social isolation, may trigger symptoms of alexithymia during the COVID-19 outbreak. Neurobiological studies have also shown that alexithymia is associated with dysregulated cortisol levels in response to stressful situations [100,101], resulting in higher anxiety and depressive symptoms [102,103]. Higher psychopathological symptoms due to COVID-19 among individuals with high levels of alexithymic have also been shown [61,62,63,64], in line with our findings.

### 4.2. Indirect Effects of Attachment to Parents and Peers via Alexithymia

Results of our mediation analyses further supported the peculiar contribution played by, respectively, attachment to mothers, fathers, and peers and their relationship with late adolescents’ alexithymia in predicting levels of peritraumatic distress due to COVID-19.

Specifically, we found that attachment to mothers and peers, but not to fathers, significantly and negatively predicted alexithymia, that in turn are predictive of youths’ peritraumatic distress due to COVID-19. These results are in accordance with the studies by Besharat and coll. [67] and Estévez and coll. [68] that have shown the predictive effect of insecure attachment on alexithymia. The quality of early relationships with attachment figures represents the main developmental environment for children’s emotional regulation and related behaviors [104,105]. Biological studies have also highlighted the significant influence of attachment in the development of neuronal structures primarily involved in emotional regulation (i.e., amygdala, and hippocampus) [106,107]. If attachment figures respond to children’s needs and emotional states with sensitivity and responsivity, individuals’ ability to regulate emotions will be enhanced by the development of a secure attachment. In contrast, relationships with parents in which caregivers show difficulties in expressing their emotions and in recognizing and validating offspring’s emotional experience, providing a secure base, lead to emotional dysregulation problems over time [108]. These difficulties in identifying and understanding the emotional states explain the risk influence exerted by insecure attachment in predicting higher levels of alexithymia [67,109], which is considered as one of the main characteristics of emotional dysregulation [110].

Interestingly, our results showed a significant direct effect of attachment to fathers on late adolescents’ peritraumatic distress. However, attachment to father was not a significant predictor of alexithymia. Conversely, although both the direct and total effects of attachment to mothers on peritraumatic distress due to COVID-19 were not significant, levels of adolescent’s alexithymia fully mediated this relationship. This result suggested that the relation between attachment to mothers and late adolescents’ mental health problems resulting from the COVID-19 outbreak is not in a simply direct linear relationship, but that alexithymia plays an important mediating role. Notably, these direct and indirect associations between parental attachment and psychopathological symptoms resulting from the COVID-19 pandemic shown that late adolescents, although in a phase of life in which they tend to become more independent from their parents [25,26], continue to count on their mothers and fathers as a secure base [91,111]. Consistent with our findings, previous studies have also shown that attachment to parents—and especially to mothers [112,113]—significantly affects adolescents’ ability to regulate their emotions. A possible explanation could be that mothers generally constitute the primary caregiver throughout the lifetime, including during young adulthood [91,114,115]. In line with this, international [116,117] and national [91,118] research has shown that young adults are more involved with their mothers than fathers, and prefer their mothers as attachment figures of support for listening to their emotional problems and for clarifying their feelings. Moreover, previous studies have also shown that youths perceived a greater asymmetry in their relationship with their fathers [119,120], experienced lower levels of intimacy with fathers [119], and felt better understood by their mothers [121]. Our findings go further in this direction, adding new knowledge on the key contribution played by the relationship with mothers in the late adolescent’s psychological response to the pandemic. Indeed, to our best knowledge, this is the first study to evidence the complex relationship existent between late adolescents’ attachment to mothers, alexithymia, and psychopathological outcomes in response to the COVID-19 pandemic, although previous studies have found that alexithymia mediated the effect of attachment to parents (especially to mothers) on adolescents’ and young adults’ psychopathology [68,70]. However, the significant direct path between attachment to fathers and peritraumatic distress due to COVID-19 confirmed the key contribution played by the quality of paternal relationship in shaping children’s psychological well-being [122,123,124], and that this influence continues to assume a crucial role for late adolescents’ psychological well-being [91].

Finally, regarding the role played by attachment to peers, direct, total, and indirect effects via alexithymia were significant, showing that alexithymia partially mediated the relation between attachment to peers and peritraumatic distress due to COVID-19. Interestingly, the size of the effects of attachment to peers’ influences was greater compared to those exerted directly and/or indirectly by attachment to parents. Overall, these findings supported the evidence that, during late adolescence/young adulthood, individuals become greater independent and autonomous from their parents and more oriented on the development of personal identity and relationships with their peers [13,125]. Although the relationship with parents continues to assume a crucial support role [126,127,128]—as our findings have also shown—attachment to peers become their essential secure base and source of emotional support [129,130,131]. Consequently, in line with our findings, during this developmental stage, insecure attachment to peers represents the strongest predictor for psychopathological problems, compared to insecure attachment to parents [132,133]. In times of the COVID-19 outbreak and its related restrictions, insecurely attached late adolescents tend to be more vulnerable than their secure peers to the psychopathological consequence of the pandemic [49], both because they are less able to manage their negative feelings (as our study has suggested) and because they would tend to seek less online interactions and emotional support from friends compared to their secure peers [134]. In fact, other studies have shown that, during the pandemic, feeling socially connected with friends via online and social media interactions helped adolescents in reducing the sense of loneliness triggered by the COVID-19-related restrictions [135,136] and associated psychopathological symptoms [137,138].

### 4.3. Possible Limitations and Strengths

There are some limitations to this study. First, this is a cross-sectional study and we have not conducted a pre-COVID-19 assessment of the study variables. Moreover, the use of cross-sectional approaches for mediation analyses may in itself produce biased results [139]. Consequently, the effects we found and assumed to be resulting from the COVID-19 pandemic should be taken with caution and confirmed by further longitudinal studies. In addition, the online convenience sampling technique that we used to collect the data limits the generalizability of the results, which should be confirmed by further studies using probability sampling techniques. In addition, we assessed late adolescents’ attachment to parents and peers, alexithymia, and peritraumatic distress due to COVID-19 through self-report instruments which, although are extensively validated and used in the field of developmental psychopathology research, should be integrated with more objective measures (e.g., clinical interviews). Notwithstanding the above limitations, this is the first study to explore the possible complex relation between adolescents’ attachment to parents and peers, alexithymia, and peritraumatic distress due to COVID-19, showing the significant mediation role played by adolescents’ alexithymia on the effects of attachment to mothers and peers on late adolescents’ psychological distress. Moreover, we considered the specific contribution exerted by late adolescents’ attachment to mothers, fathers, and peers on alexithymia and psychological response in the face of COVID-19, whereas only a few studies have distinguished effects of differential relations of parental and peer attachment on adolescent’s mental health.

### 4.4. Implications for Practice and Clinic Applications

Our findings suggest that, during late adolescence, interventions focused on the promotion of parent–adolescent and peer social relationships may prevent difficulties in facing the COVID-19 outbreak. Attachment security may support adolescents’ psychological response to the pandemic. Thus, interventions focused on parent–adolescent relationships can prevent the short- and long-term consequences of the current health crisis and improve adolescents’ adaptation in the face of possible future stressful life events. Consistent with the need to structure rapid interventions to help youths in facing the current pandemic, previous studies have shown that even brief interventions of a few sessions on the parent–child relationship can promote more sensitive parenting, a greater sense of security in children, as well as better coping strategies to face stressful life events [140,141]. Moreover, improving the quality of the parent–child relationship may promote more adaptive mental health responses in adolescents also through their positive effects on other protective factors commonly associated with the ability to cope with stress and pandemic, such as emotional regulation and alexithymia. To this end, attachment-based family therapy provided by telehealth services has shown their effectiveness in reducing adolescents’ psychopathological symptoms [142], also in the time of the COVID-19 pandemic [143]. On the other hand, our findings have highlighted the key role played by the quality of relationships with peers in contrasting the sense of loneliness resulting from the COVID-19 related restrictions and promoting a positive adaptation to the pandemic [136]. Previously, interventions focused on online peer-to-peer social support have been shown to provide a protective effect against adolescents’ psychopathological problems [144]. Helping adolescents to stay in interaction with each other, provide and seek emotional support, and share experiences, improved adolescents’ mental health. This evidence suggests that, in the context of the COVID-19 pandemic, interventions aimed at promoting online peer support are called for. In line with this, recent studies on healthcare workers have shown the effectiveness of peer support online interventions in reducing psychopathological symptoms resulting from the COVID-19 pandemic [145,146]. Our study, together with this preliminary evidence, supports the importance of implementing peer-based interventions to prevent the psychological consequence of COVID-19 on adolescents’ psychological well-being.

## 5. Conclusions

This study has shown the importance of considering the specific contribution played by attachment to mothers, fathers, and peers on psychological health during late adolescence, as well as their complex relations with adolescents’ levels of alexithymia in studying psychological consequences of the COVID-19 pandemic and the related risk and protective factors. Specifically, our findings have suggested a prominent maternal influence on late adolescents’ emotional difficulties, that in turn affected psychological responses to the COVID-19 pandemic. The relationship with fathers had also a crucial role, directly influencing children’s psychological adjustment to the COVID-19 outbreak. However, the insecure attachment to peers is shown to have the greatest impact, leading to higher levels of late adolescents’ peritraumatic distress due to COVID-10 directly than via alexithymia. These results could be informative for the planning of intervention programs more targeted in helping late adolescents to face the current health emergency and preventing emotional-behavioral problems. Specifically, treatment strategies focused on promoting peer social relationships and supportive parent–adolescent relationships, as well as the ability to recognize and discriminate one’s own and others’ emotions, may be more effective in preventing and/or reducing short- and long-term psychopathological consequences related to the COVID-19 pandemic.

## Figures and Tables

**Table 1:** Pearson correlation coefficients between the study variables.

	1.	2.	3.	4.	5.	6.	7.	8.	9.	10.	11.	12.
1. Sex	1											
2. Age	−0.06	1										
3. Educational Level	0.05	0.59 **	1									
4. Occupation	−0.02	0.16 **	0.08	1								
5. Relationship Status	0.12 **	0.17 **	0.13 **	0.14 **	1							
6. Family Status	0.03	−0.12 **	−0.14 **	0.01	−0.09 *	1						
7. Living Setup	0.11 *	−0.15 **	−0.12 *	−0.14 **	−0.04	0.06	1					
8. CPDI	0.06	−0.08	−0.16 **	−0.13 **	−0.09 *	0.10 *	0.18 **	1				
9. IPPA Mother	0.00	0.02	0.04	0.09 *	0.15 **	0.01	−0.04	−0.20 **	1			
10. IPPA Father	0.03	0.00	−0.01	0.13 **	0.13 **	0.11 *	−0.03	−0.27 **	0.46 **	1		
11. IPPA Peers	0.06	0.11 *	0.16 **	−0.01	0.06	−0.10 *	−0.05	−0.32 **	0.31 **	0.22 **	1	
12. TAS-20	0.01	−0.18 **	−0.25 **	−0.01	−0.16 **	0.11 *	0.10 *	0.44 **	−0.24 **	−0.14 **	−0.41 **	1

Note. CPDI = COVID-19 Peritraumatic Distress Index; IPPA = Inventory of Parent and Peer Attachment; IPPA mother = Attachment to mother; IPPA father = Attachment to father; IPPA peers = Attachment to peers; TAS-20 = Total scale of the Toronto Alexithymia Scale: * *p* < 0.05, ** *p* < 0.01.

**Table 2:** Results of hierarchical multiple regression analyses predicting adolescents’ peritraumatic distress due to COVID-19, controlling for sociodemographic covariates.

		Model 1			Model 2		
		*B*	*t*	*p*	*B*	*t*	*p*
**Covariates**							
Intercept			2.15	0.03 *		0.74	0.45
Age		0.08	1.48	0.13	0.09	10.71	0.09
Level of education ^a^							
	Higher school	−0.01	−0.08	0.93	0.07	0.56	0.58
	More than higher school	−0.18	−1.22	0.22	−0.02	−0.19	0.85
Occupation ^b^							
	Unemployed student	−0.06	−0.63	0.52	0.02	0.19	0.85
	Employed student	−0.12	−1.35	0.17	−0.03	−0.40	0.69
	Employed part time	0.04	0.86	0.38	0.04	0.69	0.49
	Employed full time	−0.14	−1.89	0.58	−0.11	−10.66	0.10
Relationship status ^c^							
	Partnered	−0.09	−1.94	0.52	−0.03	−0.62	0.54
	Cohabit	0.06	1.01	0.31	0.06	10.01	0.31
Family status ^d^		0.04	0.96	0.33		0.04	10.07
Living setup ^e^							
	Friends/Housemates	0.16	2.11	0.03 *	0.13	10.78	0.08
	Partner	0.02	0.26	0.79	0.07	0.92	0.36
	Family members	0.30	3.27	0.001 ***	0.23	20.76	0.01 **
Predictors							
IPPA	Attachment to Mother				0.01	0.30	0.76
	Attachment to Father				−0.19	−40.13	0.000 ***
	Attachment to Peers				−0.13	−20.73	0.01 **
TAS-20					0.34	70.40	0.000 ***
Adjusted R^2^		0.07			0.28		
R^2^ change		0.10			0.20		
F for R^2^ change		3.81 **			32.93 ***		

Note. ^a^ Less than higher school is the reference group, ^b^ Unemployed is the reference group, ^c^ Single is the reference group, ^d^ Intact family is the reference group, ^e^ Living alone is the reference group; SE= Standard error; IPPA = Inventory of Parent and Peer Attachment; TAS-20 = Total scale of the Toronto Alexithymia Scale; Coefficients shown are standardized regression coefficients. * *p* < 0.05, ** *p* < 0.01, *** *p* < 0.001.

**Table 3:** Hierarchical multiple regression model ANOVA.

Model		Sum of Squares	Df	Mean Square	F	*p*
1	Regression	9808.69	13	754.51	3.81	0.000 ***
	Residual	87015.56	440	197.76		
	Total	96824.25	453			
2	Regression	30001.95	17	1764.82	11.51	0.000 ***
	Residual	66822.30	436	153.26		
	Total	96824.25	453			

Note. *** *p* < 0.001.

**Table 4:** Indirect effects of late adolescents’ attachment to mothers, fathers, and peers on peritraumatic distress due to COVID-19 through alexithymia.

Indirect Effect	Effect (BootSE)	LLCI	ULCI
IPPA mothers → TAS-20 → CPDI	−0.04 (0.02)	−0.08	−0.01
IPPA fathers → TAS-20 → CPDI	0.01 (0.05)	−0.03	0.04
IPPA peers → TAS-20 → CPDI	−0.13 (0.02)	−0.18	−0.09

Note. IPPA = Inventory of Parent and Peer Attachment; IPPA mother = Attachment to mother; IPPA father = Attachment to father; IPPA peers = Attachment to peers; TAS-20 = Total scale of the Toronto Alexithymia Scale; CPDI = COVID-19 Peritraumatic Distress Index; BootSE = Boot-strapped standard error; LLCI = Lower-level confidence interval; ULCI = Upper level confidence interval; all bold values are statistically significant.

## Data Availability

The data presented in this study are openly available in FigShare at doi:10.6084/m9.figshare.16546488.
